# Topical Administration of Verapamil in Poly(ethylene glycol)-Modified Liposomes for Enhanced Sinonasal Tissue Residence in Chronic Rhinosinusitis: *Ex Vivo* and *In Vivo* Evaluations

**DOI:** 10.1021/acs.molpharmaceut.2c00943

**Published:** 2023-02-06

**Authors:** Maie S. Taha, Shallu Kutlehria, Anisha D’Souza, Benjamin S. Bleier, Mansoor M. Amiji

**Affiliations:** † Department of Otolaryngology, Massachusetts Eye and Ear, Harvard Medical School, Boston, Massachusetts 02114, United States; ‡ The Department of Pharmaceutical Sciences, School of Pharmacy and Pharmaceutical Sciences, Northeastern University, Boston, Massachusetts 02115, United States; § The Department of Pharmaceutics and Industrial Pharmacy, Faculty of Pharmacy, Cairo University, Cairo 11562, Egypt; ∥ The Department of Chemical Engineering, College of Engineering, Northeastern University, Boston, Massachusetts 02115, United States

**Keywords:** verapamil, chronic rhinosinusitis, liposomes, intranasal administration, sinonasal residence, muco-penetration

## Abstract

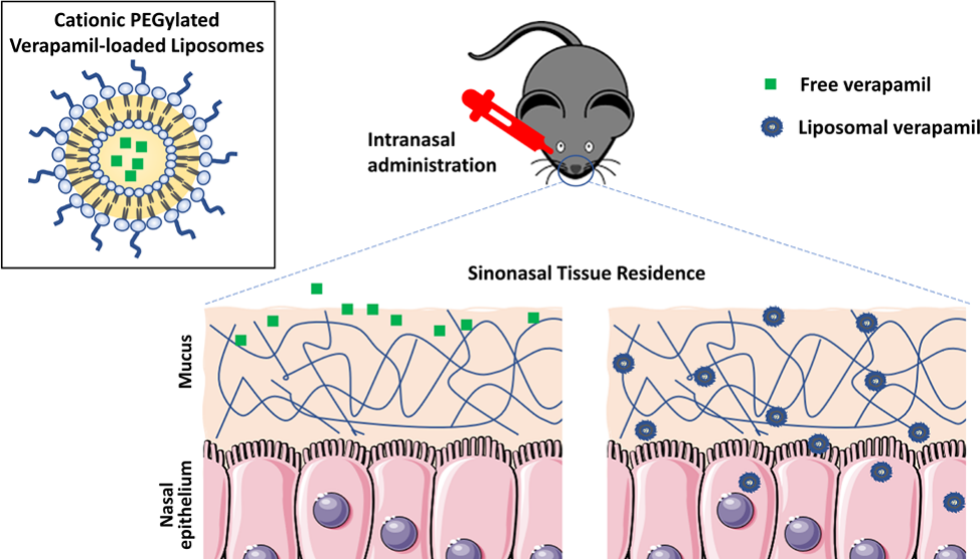

Verapamil is a calcium channel blocker that holds promise for the therapy of chronic rhinosinusitis (CRS) with and without nasal polyps. The verapamil-induced side effects limit its tolerated dose via the oral route, underscoring the usefulness of localized intranasal administration. However, the challenge to intranasal administration is mucociliary clearance, which diminishes localized dose availability. To overcome this challenge, verapamil was loaded into a mucoadhesive cationic poly(ethylene glycol)-modified (PEGylated) liposomal carrier. Organotypic nasal explants were exposed to verapamil liposomes under flow conditions to mimic mucociliary clearance. The liposomes resulted in significantly higher tissue residence compared with the free verapamil control. These findings were further confirmed in vivo in C57BL/6 mice following intranasal administration. Liposomes significantly increased the accumulation of verapamil in nasal tissues compared with the control group. The developed tissue-retentive verapamil liposomal formulation is considered a promising intranasal delivery system for CRS therapy.

## 1 Introduction

Chronic Rhinosinusitis (CRS) is an inflammatory disease affecting the sinonasal mucosa. The mechanisms underlying chronic mucosal inflammation in CRS are poorly understood. Consequently, the development of cost-effective, targeted therapies remains a significant unmet need. P-glycoprotein (P-gp) is an ATP-dependent transmembrane efflux pump that has been shown to contribute to CRS pathogenesis.^1,2^ Our group has demonstrated P-gp overexpression in the sinus epithelium of CRS patients with Type 2 inflammation.^1,2^ We have also shown its role in modulating epithelial proinflammatory cytokines secretion.^3−5^

Verapamil is a calcium channel blocker that is used for the treatment of hypertension and cardiac arrhythmias.^6^ Additionally, verapamil is known for inhibiting P-gp, a function through which it could modulate inflammatory responses in human T-cells, preclinical asthma models, and nasal polyps.^4,7−10^ Our group has demonstrated through a double-blind, placebo-controlled, randomized clinical trial that oral verapamil significantly improved both subjective and objective measures of CRS with nasal polyps (CRSwNP).^11^ The dose we could study with systemic administration was limited by safety considerations, including the potential for cardiac side effects. Given these findings, we hypothesized that intranasal verapamil delivery would allow higher local dosing, resulting in even greater efficacy while limiting systemic side effects.

Liposomes are spherical vesicles made of phospholipid bilayers with aqueous core, which can be loaded with lipophilic or hydrophilic drugs. Liposomes have been studied as intranasal drug delivery systems for either localized or systemic effects.^12,13^ Using liposomes for localized nasal drug delivery can enhance drug residence in the nasal cavity and thus maximize local efficacy while limiting systemic side effects.^14^ Fifteen to twenty minutes is the maximal residence time in the nose due to mucociliary clearance.^15^ As not all of the fluid in the nose is immediately available to the surface for drug uptake, enhancing residence time is advantageous. Positively charged liposomes have the potential to enhance tissue residence and, consequently, tissue uptake via interaction with the negatively charged mucus/ epithelium in the nasal mucosa.^16^ DOTAP is a commonly used cationic lipid reported for its safety for intranasal administration.^17,18^ Incorporating poly(ethylene glycol) (PEG) chains on the surface of liposomes would additionally enhance mucus penetration as a strategy to overcome mucociliary clearance and increase localized residence time.^19^ The hydrophilicity and flexibility of PEG chains enable its diffusion through the mucus mesh; thus, PEG coatings have been previously used to obtain mucus-penetrating nanoparticles.^19−21^

The use of vesicular systems for systemic delivery of verapamil via the intranasal route has been reported scarcely before. Verapamil enclosed in chitosan-composite transferosomes permeated efficiently through nasal sheep mucosa.^22^ When tested in a preclinical animal model, it resulted in a 6.3-fold increase in bioavailability compared to oral solution.^22^ In another preclinical study, verapamil-loaded chitosan microspheres showed 4.5-fold increase in systemic bioavailability relative to oral verapamil.^23^ To the best of our knowledge, the use of nanoparticle systems to increase verapamil intranasal residence for localized therapy purposes has not been reported before.

The purpose of this study was therefore to load verapamil into a PEGylated liposomal formulation and further test its effectiveness in increasing its sinonasal residence time. The enhanced tissue residence was evaluated *ex vivo* using nasal tissue explants and *in vivo* in C57BL/6 mice following intranasal administration.

## 2 Materials and Methods

### 2.1 Materials

Verapamil hydrochloride, cholesterol, and CelLytic MT cell lysis reagents were purchased from Sigma-Aldrich (St. Louis, MO). The lipids 1,2-dioleoyl-3-trimethylammonium-propane (chloride salt) (DOTAP) and 1,2-distearoyl-*sn*-glycero-3-phosphoethanolamine-*N*-[amino(polyethylene glycol)-2000] (ammonium salt) (DSPE-PEG) were purchased from Avanti Polar Lipids. Bronchial epithelial growth medium (BEGM) was purchased from Lonza (Basel, Switzerland). Pierce BCA Protein Assay Kit was purchased from Thermo Fisher Scientific (Waltham, MA). Solvents were of HPLC (high-pressure liquid chromatography) grade and were purchased from Fisher Scientific.

### 2.2 Preparation of Verapamil-Loaded PEGylated Liposomes

Liposomes were prepared by thin-film hydration method.^13^ Briefly, 1 mL of stock solution of each of the lipids DOTAP (16.76 mg), Cholesterol (3.09 mg), DSPE-PEG 2000 (1.11 mg) in chloroform (molar ratio 6:2:0.1) was mixed with 1 mL of verapamil (2 mg/mL) in methanol in a round-bottom flask. The solvents were evaporated using a rotary evaporator (RV Control 10, IKA, Staufen, Germany) rotating at 100 rpm and 37 °C to form a uniform thin lipid film. The flask was then kept in vacuum overnight for complete removal of residual solvents. The film was hydrated by adding 1 mL of phosphate-buffered saline (PBS) and vortexing till a white dispersion was formed. This was followed by five repetitive freeze–thaw cycles by placing the flask with dispersion in an ice bath and warm water bath, alternatingly, for 2 min each, separated by vortexing for 30 s. The liposomal dispersion was then probe-sonicated on ice for 5 min, followed by centrifugation at 14,000 rpm for 15 min at 4 °C using ultra centrifugal filters of 3 kDa molecular weight cut off (Amicon Ultra-2, Millipore Sigma, Burlington, MA). The concentrated liposomal dispersion was collected and used for all experiments.

### 2.3 Characterization of the Liposomal Formulation

The prepared liposomes were characterized for particle size, polydispersity index (PDI), and zeta potential via dynamic light scattering (DLS) using a Malvern Zetasizer Nano ZS90 (Worcestershire, U.K.). For DLS analysis, liposomes were diluted to 1 mg/mL concentration in 10 mM phosphate buffer (pH 7.4), and the analysis was done at 25 °C. Verapamil loading content was estimated by suspending a certain weight of liposomes in acetonitrile and bath sonicating for 30 min to disrupt them. The suspension was then centrifuged to obtain a clear supernatant that was analyzed for its verapamil concentration using a high-pressure liquid chromatography (HPLC) method modified from a previous study.^24^ For determining the encapsulation efficiency (EE), the liposomes were centrifuged at 14,000*g* for 15 min at 4 °C using ultra centrifugal filters of 3 kDa molecular weight cut off (Amicon Ultra-2, Millipore Sigma, Burlington, MA). The filtrate (containing free drug) was collected and analyzed using HPLC. The EE was calculated by subtracting the amount of free drug from the total drug feed used for liposome preparation. The percentage EE was calculated using the equation %EE = [(Total drug used – Free drug)/Total drug used] × 100. HPLC analysis was performed using a Waters Alliance 2695 HPLC System equipped with a Microsorb-MV C18 column (5 μm particle size, 250 × 4.6 mm). The injection volume was 20 μL, and the mobile phase was composed of acetonitrile and 20 mM potassium dihydrogen phosphate (pH made to 3 with phosphoric acid) and was run in an acetonitrile gradient of 40–50% over 20 min at 1 mL/min (at 0–10 min: 40% of A and 60% of B, and at 10–20 min: 50% of A with 50% of B). Verapamil was detected using a UV detector at a wavelength of 235 nm. The liposomes were imaged by the JEOL JEM-1000 transmission electron microscope (JEOL Ltd., Tokyo, Japan) after negative staining with 1.5% phosphotungstic acid.

### 2.4 *In vitro* Verapamil Release from Verapamil-Loaded Liposomes

*In vitro* release studies of verapamil from verapamil-loaded liposomes were carried out in simulated nasal fluid (SNF) using the dialysis bag diffusion method.^25^ Briefly, liposomes (or verapamil solution as a control) equivalent to 0.5–0.6 mg of verapamil were placed into a regenerated cellulose dialysis tubing membrane with a MWCO 12 kDa (Pur-A-Lyzer Mini, Sigma-Aldrich, Milwaukee, WI). The sample-loaded and capped Pur-A-Lyzer tubes were immersed into glass vials containing 6 mL of the dissolution medium (SNF). SNF is composed of an aqueous solution of 7.45 mg/mL NaCl, 1.29 mg/mL KCl, and 0.32 mg/mL CaCl_2_·2H_2_O with a pH maintained between 6.2 and 6.8. The vial was placed in a water bath, maintained at 37 °C, and shaken at 100 rpm. Dissolution medium was withdrawn (samples of 300 μL) at predetermined time points of 0.25, 0.5, 1, 2, 4, 12, and 24 h from outside the dialysis bag. The absorbances of the collected aliquot were determined at 278 nm on UV–visible spectrometer using a plate reader (Biotek, Winooski, VT), and the amount of verapamil released from the liposomes was calculated.

### 2.5 Primary Human Turbinate Tissue Sampling

Human sinonasal tissue sampling was approved by the Massachusetts Eye and Ear Infirmary’s and Northeastern University’s Institutional Review Boards (IRB number: 2019P001204). All de-identified tissue samples were taken from patients who had not been exposed to antibiotics or steroids for at least 4 weeks. Inclusion criteria included healthy patients undergoing turbinate reduction surgery for noninflammatory disease. Exclusion criteria included patients with ciliary dysfunction, autoimmune disease, cystic fibrosis, immunodeficiency and smoking, presence of allergy, and asthma. Diagnostic criteria for asthma, aspirin-exacerbated respiratory disease (AERD), and allergic rhinitis were based on both clinical history and allergy testing.

### 2.6 Verapamil Uptake and Retention in Nasal Tissue Explants

Harvested turbinates were immediately sectioned into 5-mm explants using a standard biopsy punch (Integra Meltex), taking care to maintain an intact epithelial layer in each explant, as previously described.^3^ Explants were individually placed in the wells of a cell culture chamber (QV900, Kirkstall Ltd, U.K.) with 2 mL of bronchial epithelial cell growth medium (BEGM) per well. BEGM (30 mL) containing either dissolved (free) verapamil or verapamil-loaded liposomes was flowed through the chamber wells for 30 min. The verapamil concentration was set as 74 μg/mL. The chamber was incubated at 37 °C and 5% CO2. After 30 min, the explants were rinsed with PBS to remove any surface-bound drug or liposomes and kept at −80 °C for later analysis of the verapamil content. For another set of similarly treated explants, fresh verapamil-free BEGM was allowed to flow through the chamber for 60 min as a washout period. At the end of the washout period, explants were rinsed with PBS and stored at −80 °C for later analysis of the verapamil content.

Explants were homogenized in 400 μL of cell lytic buffer, and the homogenates (200 μL) were spiked with 1 μL of propranolol hydrochloride (100 μg/mL) aqueous stock, used as an internal standard for extraction. The spiked homogenates were extracted with 800 μL of ethyl acetate by vortexing for 15 min. The organic layer was separated by centrifugation at 4000 rcf for 10 min at 4 °C, and 600 μL of it was transferred to new tubes and dried in air. Dried films were reconstituted in 75 μL of mobile phase and analyzed for verapamil concentration using HPLC. Standards for HPLC analysis were prepared by spiking tissue homogenate (from the same patient’s tissue) with verapamil (from standard stocks) to final concentrations of 5, 2.5, 1.25, 0.63, 0.13, 0.08, and 0.04 μg/mL and were similarly processed as the samples. The remainder of the tissue homogenates were centrifuged at 13,000 rcf for 20 min at 4 °C, and supernatants were collected for protein quantification using a BCA assay.

### 2.7 Sinonasal Bioavailability of Intranasally Administered Verapamil-Loaded Liposomes

#### 2.7.1 Administration and Tissue Collection

The study design was based on guidelines and approval of the Institutional Animal Care and Use Committee (IACUC) of Northeastern University (IACUC protocol number: 19-0208R-A8). Male C57BL/6 mice (5–6 weeks old, ∼20–25 g) were purchased from Charles River Laboratories (Wilmington, MA). After 1 week of acclimatization, the mice were treated with verapamil-loaded liposomes or verapamil solution (at a verapamil-equivalent dose of 1.7 mg/Kg, therefore total dose administered was 0.034–0.043 mg of verapamil) instilled in both nostrils (10–12 μL/nostril). Each treatment was administered to 24 mice. At predetermined time points (0.5, 1, 2, 4, 12, and 24 h post-treatment), 4 mice were randomly selected for nasal tissue collection from each group. The mice were euthanized, and nasal tissues from both right and left passages were harvested and stored at −80 °C until analysis.

#### 2.7.2 Analysis of Verapamil in Nasal Tissue

The mice’s nasal tissues were weighed and incubated with 0.1 N NaOH (90 μL/20 mg tissue). The tissues were homogenized, spiked with 0.1 μg of propranolol hydrochloride as an internal standard, and then extracted with 300 μL of diethyl ether by vortexing for 5 min. After centrifugation at 4000 rcf for 15 min at 4 °C, 200 μL of diethyl ether was separated and left to dry in air. Dried films were resuspended in 100 μL of mobile phase and analyzed with HPLC as described above (Section 2.3).

### 2.9 Statistical Analysis

All data are presented as means ± standard deviation. Statistical analyses were performed with GraphPad Prism 8 (La Jolla, CA). Data were analyzed by two-tailed student *t*-test or Two-way ANOVA with Sidak’s multiple comparisons test, as indicated. A *p*-value of <0.05 was considered statistically significant.

## 3 Results

### 3.1 Preparation and Characterization of Verapamil-Loaded Liposomes

**Figure 1:**
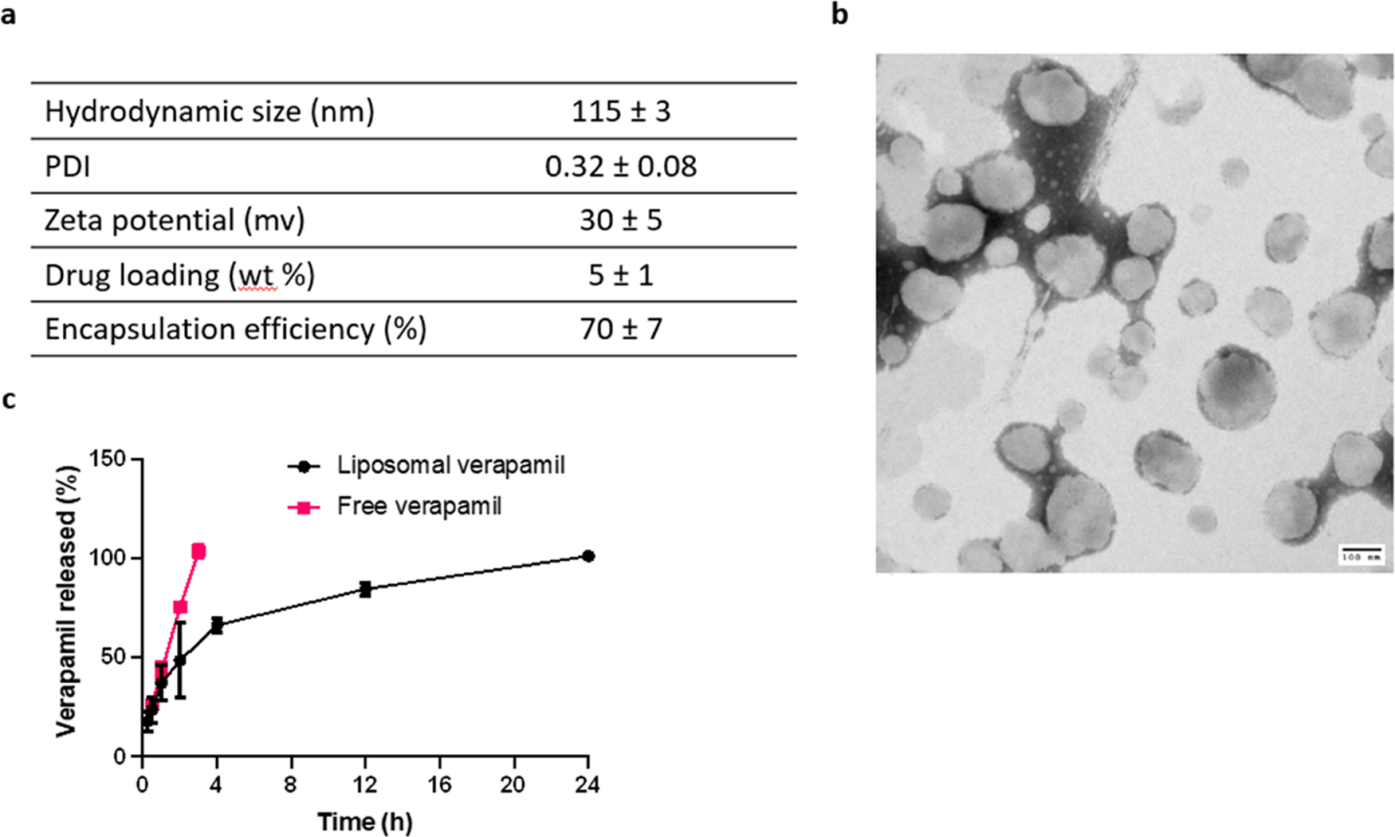
Characteristics of verapamil-encapsulated poly(ethylene glycol)-modified (PEGylated) liposomes. (a) Characterization of the verapamil-loaded liposomes (*n* = 3, values represented as mean ± SD), (b) representative transmission electron microscopy image of the prepared PEGylated liposomes (scale bar = 100 nm), and (c) *in vitro* release profile of verapamil from verapamil-loaded liposomes (*n* = 3), values represented as mean ± SD.

Verapamil-loaded PEG-modified liposomes were prepared using thin-film hydration. The lipid film was composed of DOTAP (a cationic lipid), cholesterol (a bilayer stabilizer), and DSPE-PEG at a 6:2:0.1 molar ratio corresponding to 74, 25, and 1 mol %, respectively (Table S1). The z-average of verapamil liposomes, as measured using DLS, was 115 ± 3 nm (Figure 1a, Supporting Information Figure 1). Their surface charge was measured to be 30 ± 5 mV (Figure 1a), reflecting the positive charge imparted by DOTAP. The inclusion of PEG on the liposomes surface was indicated by the decrease in surface charge in comparison with liposomes prepared without DSPE-PEG (38 ± 2 mV). The TEM images confirmed the particle size and the spherical structure of liposomes showing no aggregation (Figure 1b, Supporting Information Figure 2). The liposomes had a high verapamil content of 5 ± 1 wt % and an encapsulation efficiency of 70 ± 7% (Figure 1a). Liposomal encapsulation expectedly reduced the rate of verapamil release, where 100% of the loaded verapamil was released after 24 h (Figure 1c). Diffusion of verapamil from the dialysis bag was not rate-limiting, as shown by the free verapamil control (Figure 1c).

### 3.2 Verapamil Uptake and Retention in Nasal Tissue Explants

**Figure 2:**
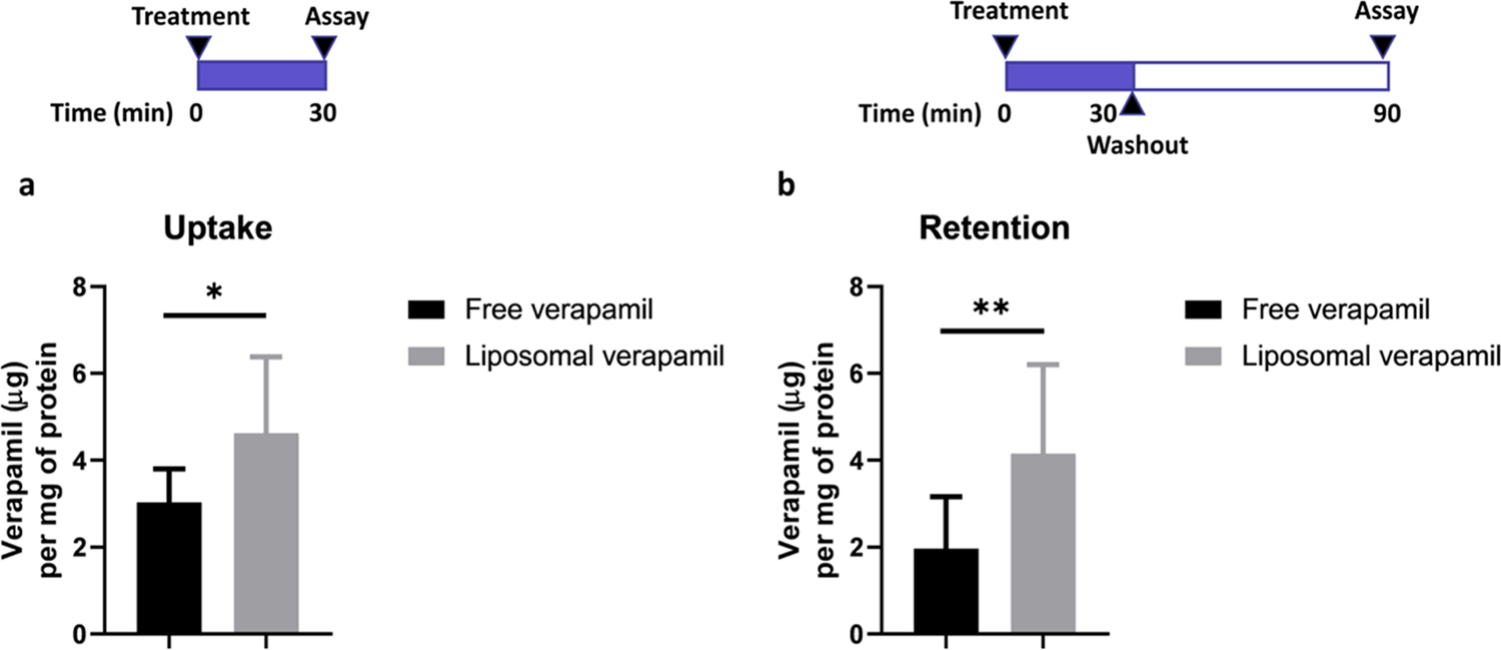
Verapamil level in organotypic turbinate explants after exposure to either free or liposomal verapamil (under flow conditions): (a) for 30 min at 74 μg/mL verapamil equivalent (*n* = 9) and (b) for 30 min at 74 μg/mL verapamil equivalent, followed by 60 min of washout period in verapamil-free media (*n* = 11). *p < 0.05 and **p < 0.01 with unpaired two-tailed *t*-test.

The ability of liposomes to enhance tissue residence of loaded verapamil was tested *ex vivo* in nasal turbinate tissue explants. To mimic the dynamic conditions associated with mucociliary clearance that can reduce liposomal residence following intranasal administration, a flow chamber was used for this experiment. Verapamil-loaded liposomes or free verapamil (i.e., solution) were incubated with turbinate explants freshly harvested from human volunteers, in the flow chamber. Liposomes significantly improved verapamil tissue uptake (1.6-fold) as compared to unencapsulated verapamil (Figure 2a) (*p* < 0.05, unpaired two-tailed *t*-test). Additionally, verapamil-loaded liposomes displayed significantly higher tissue retention as evidenced by verapamil tissue level after a 60 min washout period (Figure 2b) (*p* < 0.01, unpaired two-tailed *t*-test). Over 1 h, liposomal verapamil was maintained in the turbinate tissue (Supporting Information Figure 3b), while free verapamil was cleared to an apparently greater extent (Supporting Information Figure 3a) (90 versus 68% reduction in verapamil retention, respectively); however, statistical significance was not observed.

### 3.3 Sinonasal Bioavailability of Intranasally Administered Verapamil-Loaded Liposomes

**Figure 3:**
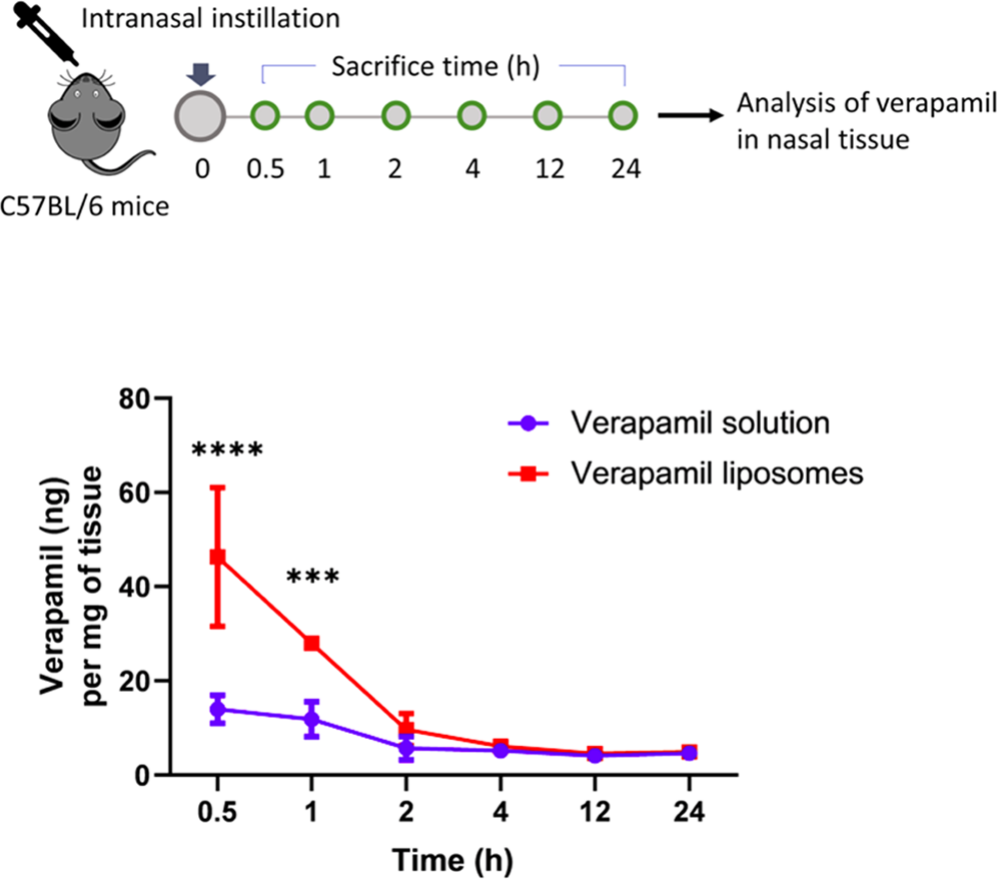
Verapamil levels over time in nasal tissues of naïve C57BL/6 mice. Mice were dosed once intranasally with verapamil liposomes and control (free verapamil) formulations at a verapamil-equivalent dose of 1.7 mg/Kg. Data represented as mean ± SD, *n* = 4 animals/time/group, ***p = 0.0001 and ****p < 0.0001, Two-way ANOVA with Sidak’s multiple comparisons.

Given the enhanced uptake and retention of verapamil in nasal tissue explants mediated by liposomes, we next examined the pharmacokinetics of verapamil-loaded liposomes in a murine model. Naïve C57BL/6 mice were treated with a single intranasal instillation of verapamil-loaded liposomes or verapamil solution at the dose equivalent to verapamil 1.7 mg/Kg. When nasal tissues were harvested and analyzed at various time points post-administration, the verapamil levels were significantly higher with liposomes than with the control group through 1 h post-administration (Figure 3). Liposomal verapamil levels at 30 min and 1 h post-administration were 5.7 and 2.8 times, respectively, higher compared to the control group (*p* < 0.0001 and *p* = 0.0001, respectively, Two-way ANOVA with Sidak’s multiple comparisons test). These levels corresponded to 2.65 ± 0.84% and 1.6 ± 0.06% of the administered dose at 30 min and 1 h, respectively, with liposomal verapamil, versus 0.8 ± 0.17% (at 30 min) and 0.67 ± 0.21% (at 1 h) with free verapamil (Supporting Information Figure 4).

## 4 Discussion

Intranasally administered drugs are rapidly cleared from the nasal cavity toward the throat due to mucociliary clearance, leaving a very short window for their absorption.^26^ In specific, hydrophilic drug molecules such as verapamil are more prone to clearance by mucociliary activity as they inherently possess low permeability through nasal epithelial cells.^27^ In that regard, loading such drugs into liposomes that can adhere to and penetrate the nasal mucosa can improve the hydrophilic drug bioavailability in the nasal tissue. The nasal epithelium is covered by a mucus blanket, which forms a barrier against tissue contact and penetration. This mucus layer has a strong net negative charge owing to the sialic acid and sulfate content of the mucin glycoproteins.^28^ Accordingly, positively charged liposomes have the potential to enhance tissue residence and, consequently, tissue uptake via interaction with the negatively charged mucus/epithelium in the nasal mucosa. Several studies have utilized positively charged liposomes to achieve nose-to-brain drug delivery on the basis of extended residence time and uptake in the nasal epithelium.^16,29,30^ In a study by Sallam et al., triamcinolone acetonide, a hydrophobic corticosteroid, was encapsulated in three different types of nanoparticles: nanoemulsion, nanostructured lipid carrier, and polymeric oil core nanocapsules, intended for intranasal therapy of allergic rhinitis.^31^ The nanocapsules that possessed a positive surface charge due to Eudragit RS-100 present in the shell offered ∼4-fold higher nasal tissue retention compared to the other two systems that possessed negative surface charges. For loading the hydrophilic verapamil HCl, a liposomal system would be more advantageous than the polymer oil core nanocapsules, as it offers a large aqueous core volume for enhanced encapsulation efficiency. Liposomes are also well known for their drug-loading stability, especially for hydrophilic molecules where the lipid shell prevents the encapsulated drug from rapid clearance.^32^ In our study, to prepare positively charged liposomes, we used DOTAP, a cationic lipid reported for its safety for intranasal administration.^17,18^ In a clinical study on cystic fibrosis patients, 2.4 mg of DOTAP was applied to the nasal epithelium.^18^ There was no histological evidence of inflammation in the nasal biopsies examined as well as no increase in circulating inflammatory markers.^18^ For further enhancing mucus penetration and delivery to the underlying epithelium, we included PEG on the liposomes surface. The hydrophilicity and flexibility of PEG chains enable its diffusion through the mucus mesh; thus, PEG coatings have been previously used to obtain mucus-penetrating nanoparticles.^19−21^ In particular, Lai et al. have shown the superiority of PEGylated polystyrene nanoparticles in penetrating through the viscoelastic mucus collected from the sinuses of CRS patients (CRSM) compared with non-PEGylated counterparts.^33^ By analyzing trajectories of fluorescently labeled nanoparticles through CRSM, their effective diffusivities compared to those in water were calculated. While the non-PEGylated nanoparticles were slowed in CRSM by 2300-fold, PEGylated counterparts of equivalent size were only slowed by 20-fold. This muco-penetrating ability was validated at different particle sizes as well. Typically, PEGylated liposomes are prepared via the pre-insertion method, where PEG-conjugated lipid is included in the lipid composition prior to lipid film formation.^34^ The inclusion of PEG on the liposomes surface can be inferred by the reduction in surface charge due to its screening effect.^35,36^

The prepared liposomal formulation encapsulated verapamil at a 5%w/w loading content. The release profile of verapamil-loaded liposomes was biphasic, with a burst effect over the first 4 hours, followed by a slow release till the end of the study. Yang et al. reported a burst release of approximately 40% of encapsulated verapamil within the first 0.5 h, followed by a sustained release of up to 78% over the next 8 h.^25^ Similarly, the water-soluble 5-fluorouracil encapsulated in liposomes showed a burst release followed by a sustained release over 12 h.^37^

We first validated the ability of the prepared liposomes to enhance tissue residence of loaded verapamil utilizing nasal turbinate tissue explants. Nasal mucosal organotypic explants are known to maintain the tissue architecture and function and are thus used to study epithelial morphology, P-gp activity, cytokines secretion, lymphocyte infiltration, and ciliary function.^3−5,38−40^ Thus, by incubating liposomes with the freshly harvested organotypic explants, they were in direct interaction with the secreted mucus layer. We elected to perform the test in a flow chamber to conservatively simulate the fluid environment caused by nasal secretions. The unidirectional flow of liposomes (suspended in BEGM) on top of the explants would mimic mucociliary clearance, naturally impeding the liposomes deposition on the nasal tissue upon intranasal administration. Encapsulating verapamil in liposomes allowed for significantly higher tissue uptake and retention than verapamil solution. This finding confirms the importance of utilizing the mucoadhesive nanoparticulate system for delivering verapamil locally to the nasal epithelium.

We next sought to confirm the *ex vivo* retention results in a murine model of naïve C57BL/6 mice. Animals were treated with a single intranasal instillation of verapamil-loaded liposomes or verapamil solution at the dose equivalent to verapamil 1.7 mg/Kg. This dose was selected based on a previous phase 1 tolerability study of intranasal topical verapamil done by our group in CRS patients.^41^ We demonstrated that the intranasally administered liposomes significantly increased verapamil retention in the nasal tissues compared to the control group of free verapamil. The enhanced retention of liposomal verapamil in the nasal tissue experienced in the early time points (0.5 and 1 h (Figure 3)) following intranasal administration proves that it successfully overcomes the mucociliary barrier, which typically wipes particulate matter within 20 min.^15^ The use of intranasally administered liposomes as drug-free therapy for allergic rhinitis, rhinoconjunctivitis, and CRS has been studied before.^42,43^ A liposomal nasal spray improved sinusitis symptoms and rhinoscopy scores in 60 CRS patients to the same extent as topical glucocorticoid therapy administered to 30 CRS patients.^43^ In 47.8% of the patients treated with the liposomal nasal spray, the onset of action occurred within 30 min of administration, in a good correlation with our observed results of enhanced localized liposomal verapamil accumulation. The efficacious property of liposomes was attributed to their ability to integrate in the cell membrane and stabilize the nasal mucosa. This property of liposomes as a drug delivery device is of dual benefit, where it can deliver its cargo drug to the nasal mucosa with high concentration and yet avoid disturbing the mucosal barrier. On the contrary, in the case of verapamil delivery for CRS therapy, the liposomal carrier can act synergistically in alleviating symptoms.

Our previous clinical trial has demonstrated that oral verapamil significantly improved clinical indices of CRSwNP;^11^ however a limitation of this trial was the low dose used to avoid cardiac side effects of verapamil. This highlights the critical need for formulating verapamil into tissue-retentive liposomes for intranasal administration to enhance localized dose availability while limiting systemic side effects. Our developed formulation successfully augmented the localized nasal availability of verapamil and thus potentially diminished its systemic exposure. In future studies, full biodistribution profiles of free versus liposomal verapamil following intranasal administration will be explored to uncover their differential accumulation in different body organs. And importantly, based on our findings, we plan for testing the preclinical effectiveness of the developed system in a relevant murine model.

## 5 Conclusions

In this study, verapamil was loaded into a mucoadhesive cationic PEGylated liposomal carrier. The liposomes had a size of 115 nm, a surface charge of 30 mV, and a verapamil content of 5 wt %. The liposomes resulted in significantly higher tissue residence compared with the free verapamil control when tested in organotypic nasal explants under flow conditions to mimic mucociliary clearance. When intranasally administered to C57BL/6 mice, the liposomes significantly increased the accumulation of verapamil in nasal tissues compared with the control group. The developed tissue-retentive verapamil liposomal formulation is considered a promising intranasal delivery system for CRS therapy.

## Supporting Information Available

Molar composition, size distribution graphs, and TEM images of the liposomes. Verapamil level in organotypic turbinate explants after exposure to either free or liposomal verapamil. Percentage of verapamil from total administered dose in nasal tissues of C57BL/6 mice dosed intranasally with verapamil liposomes and control (PDF)
