# Supplementary material for: Topical Administration of Verapamil in Poly(ethylene glycol)-Modified Liposomes for Enhanced Sinonasal Tissue Residence in Chronic Rhinosinusitis: Ex Vivo and In Vivo Evaluations
Source: Mol Pharm. 2023 Feb 6;20(3):1729–36. doi: 10.1021/acs.molpharmaceut.2c00943 (PMC10629233; doi:10.1021/acs.molpharmaceut.2c00943)
Supplement: Supplementary file 1 — mp2c00943_si_001.pdf [file mp2c00943_si_001.pdf]

Supporting Information

Title: Topical Administration of Verapamil in Poly(ethylene glycol)-Modified Liposomes for Enhanced Sinonasal Tissue Residence in Chronic Rhinosinusitis: *Ex Vivo* and *In Vivo* Evaluations

Authors list:

Maie S. Taha, PhD,[1,2,3], Shallu Kutlehria, PhD,[2], Anisha D'Souza, PhD,[1,2], Benjamin S Bleier, MD, FACS,[1], Mansoor Amiji, PhD,[2,4*]

[1] Department of Otolaryngology, Massachusetts Eye and Ear, Harvard Medical School, Boston, MA, 02114, United States of America.

[2] The Department of Pharmaceutical Sciences, School of Pharmacy and Pharmaceutical Sciences, Northeastern University, Boston, MA, 02115, United States of America.

[3] The Department of Pharmaceutics and Industrial Pharmacy, Faculty of Pharmacy, Cairo University, Cairo, 11562, Egypt.

[4] The Department of Chemical Engineering, College of Engineering, Northeastern University, Boston, MA, 02115, United States of America.

***Corresponding author:**

Tel. 617-373-3137, Email: m.amiji@northeastern.edu

Running title:

Verapamil-Encapsulated PEGylated Liposomes for CRS

Table S1. Molar composition of verapamil-loaded liposomes.

Composition (mol%)			Verapamil (mol% from total lipids)
DOTAP	Cholesterol	DSPE_PEG (2000)	
74	25	1	25

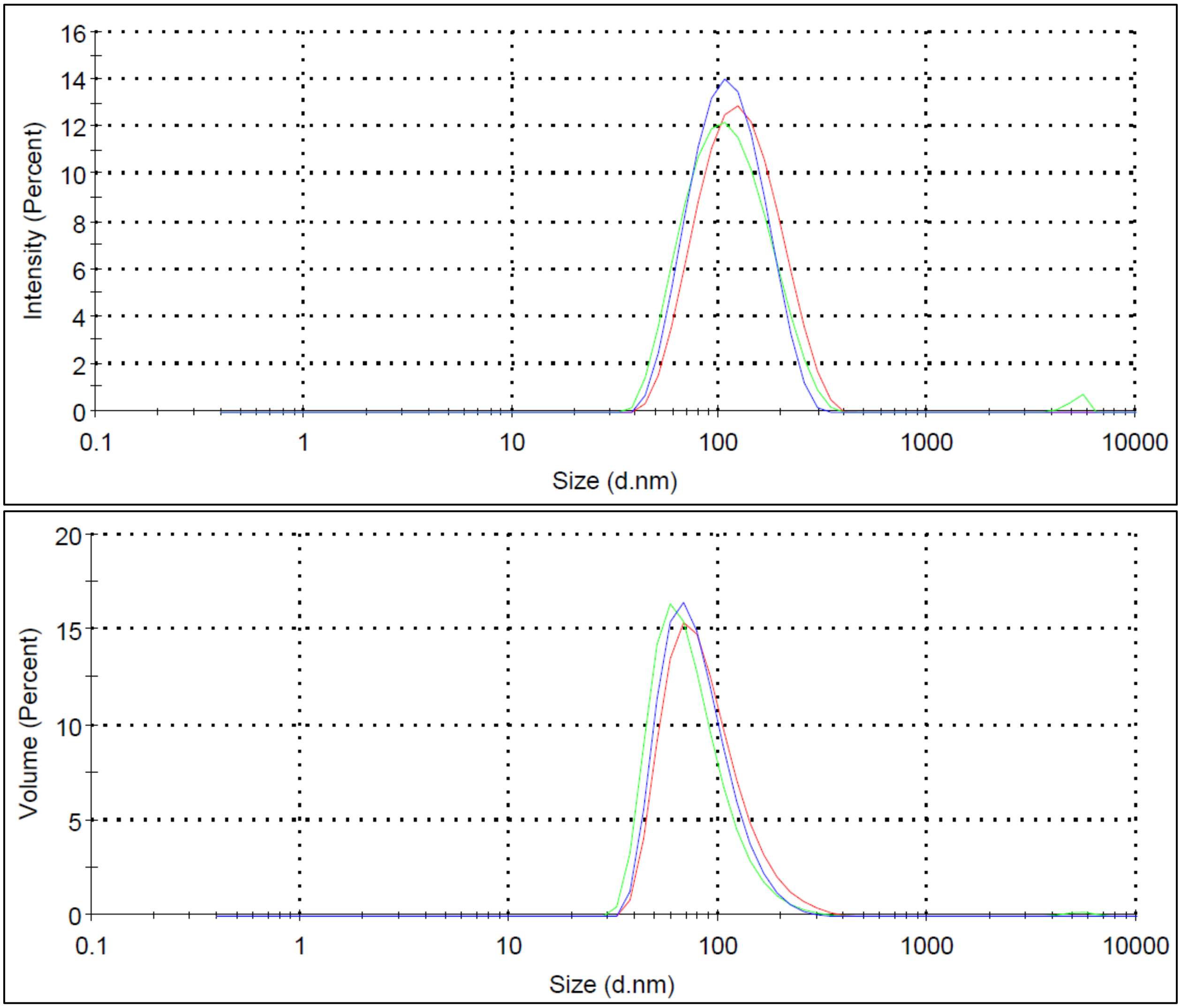

Supporting fig. 1: Size distribution graphs (by intensity and volume) for the prepared liposomes.

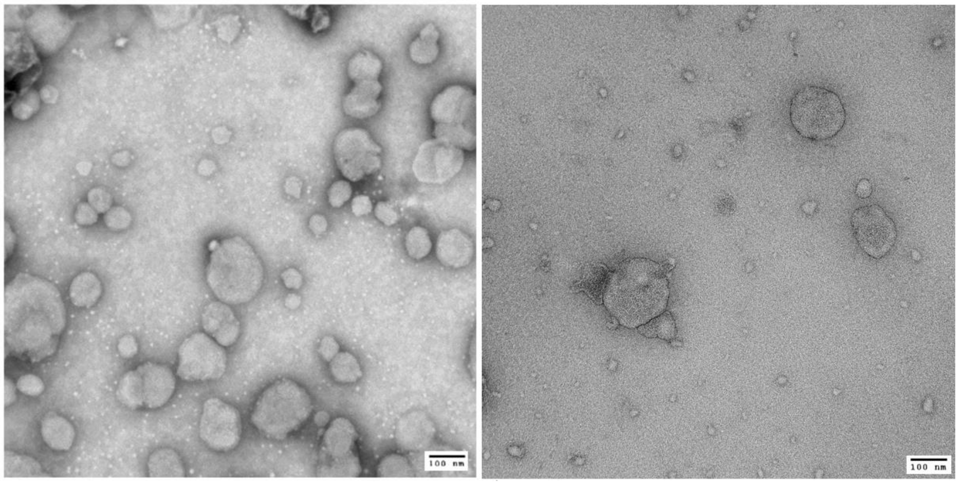

Supporting fig. 2: TEM images of the Pegylated liposomes (Scale bar = 100 nm)

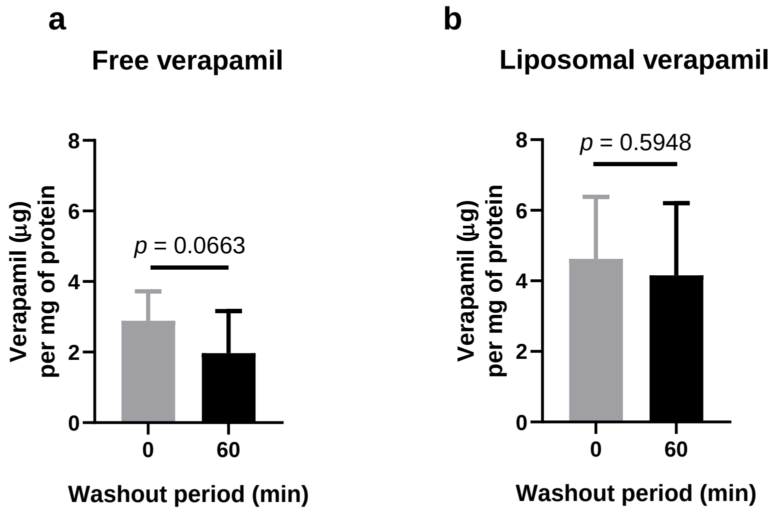

Supporting fig. 3: Verapamil level in organotypic turbinate explants after exposure to either (a) free or (b) liposomal verapamil under flow conditions for 30 min at 74 µg/mL verapamil equivalent followed by 60 min of washout period in verapamil-free media. N=9-11, statistical test used is unpaired two-tailed t-test.

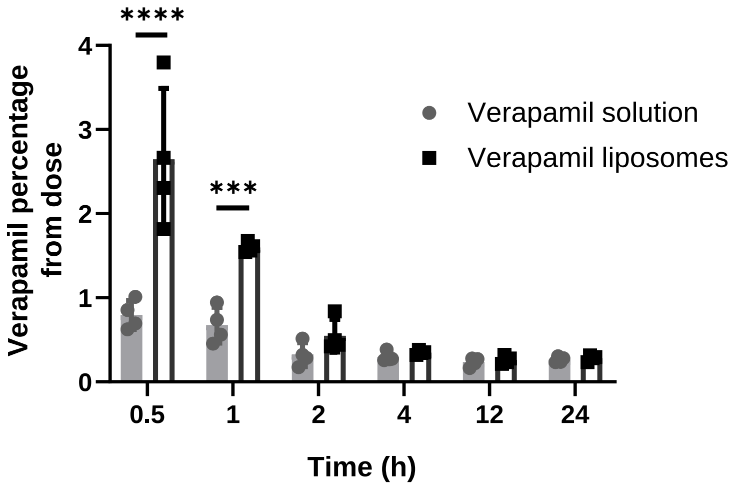

Supporting fig. 4: Percentage of verapamil from total administered dose over time in nasal tissues of naïve C57BL/6 mice dosed intranasally with verapamil liposomes and control (free verapamil) formulations at a verapamil-equivalent dose of 1.7 mg/Kg. Data represented as mean ± SD, n=4 animals/time/group, ***, $p = 0.001$ and ****, $p < 0.0001$, Two-way ANOVA with Sidak's multiple comparisons.
